# Cryo-EM single-particle analysis expanding towards increasingly native samples

**DOI:** 10.1107/S2059798325008332

**Published:** 2025-10-23

**Authors:** Jonas Moecking, Tzviya Zeev-Ben-Mordehai

**Affiliations:** a Bijvoet Centre for Biomolecular Research Utrecht University 3584 CGUtrecht The Netherlands; Diamond Light Source, United Kingdom

**Keywords:** single-particle analysis, cryo-EM, cryo-ET

## Abstract

The explosion of cryo-electron microscopy (cryo-EM) over the last decade has brought with it a range of new approaches for gaining high-resolution structural information on previously inaccessible biological systems. Cryo-EM single-particle analysis (SPA) approaches typically entail overexpression and purification of the target protein. Larger and more complex molecular assemblies often require extensive optimization of the expression, purification and reconstitution procedures. Additionally, prior knowledge of the composition of the structure of interest is required. Approaches employing cryo-focused ion beam (FIB) milling and cryo-electron tomography (cryo-ET) have proven incredibly useful for exploring protein structures within cells while maintaining near-native conditions. Such strategies avoid purification of the target protein or protein complex, yet are often still limited in throughput and achievable resolution. Here, we highlight recent studies demonstrating that the range of samples suitable for SPA is expanding towards increasingly more native samples. We specifically focus on studies investigating complex macromolecular assemblies where tailored sample-preparation strategies made them amenable for SPA, while still keeping them in close-to-native conditions. These examples show that SPA has become a discovery tool for *de novo* protein identification and complex stoichiometry in more complex and thicker samples.

## Abstract 2

Cryo-EM single-particle analysis has revolutionized structural biology by allowing high-resolution analysis of large molecular assemblies that are not amenable to crystallography or NMR. Combination of this technology with innovative sample-preparation protocols further expands the range of potential targets.

## 1. Introduction

The development and introduction of direct electron detectors for cryo-electron microscopy (cryo-EM) marked a significant milestone for structural biology (Kühlbrandt, 2014 ▸; McMullan *et al.*, 2016 ▸). Combined with advances in image-analysis software, the road was paved for protein structures to be determined routinely at near-atomic resolution by single-particle analysis (SPA) cryo-EM (Scheres, 2012 ▸; Li *et al.*, 2013 ▸; Liao *et al.*, 2013 ▸; Allegretti *et al.*, 2014 ▸; Amunts *et al.*, 2014 ▸; Kühlbrandt, 2014 ▸). Making large proteins and complexes, membrane proteins, viruses and polymers accessible to high-resolution structure determination, SPA became prominent in the toolbox of structural biologists (Nwanochie & Uversky, 2019 ▸; Glaeser *et al.*, 2021 ▸; Saibil, 2022 ▸). Moreover, SPA provides an invaluable resource to further train computational structure-prediction algorithms (*AlphaFold*, *RoseTTAFold*), which will in turn help to identify and model unknown protein structures (Jumper *et al.*, 2021 ▸; Baek *et al.*, 2021 ▸).

Samples for SPA cryo-EM need to fulfil several requirements to allow high-resolution reconstruction. As for NMR and crystallography, the target proteins for SPA must be expressed and purified before they can be applied to an EM grid, although for SPA only small sample volumes are required. Further requirements include particle abundance (or concentration), purity of the particles, conformational homogeneity, an even distribution of particles while avoiding the air–water interface, and a heterogeneous population of orientations on the EM grid (Glaeser *et al.*, 2021 ▸; Saibil, 2022 ▸). Additionally, SPA is still only applicable for proteins of >50 kDa, ideally even >100 kDa, if high-resolution structure determination is the goal (Guaita *et al.*, 2022 ▸). Satisfying all of these requirements and producing high-quality grids for data collection often demands extensive optimization and can drastically limit the achievable resolution. In addition, ice thickness can have a tremendous influence on the achievable resolution due to the limited mean free path of electrons (∼280 nm for vitrified free vesicles) and the significantly reduced signal-to-noise ratio for samples thicker than the mean free path (Grimm *et al.*, 1996 ▸; Rice *et al.*, 2018 ▸; Yesibolati *et al.*, 2020 ▸). Typically, samples for SPA are prepared with an ice thickness in the range of 10–100 nm, but improvements in detectors and the application of energy filters now also allow structure determination from thicker samples of up to ∼500 nm in benchmarking experiments (Neselu *et al.*, 2023 ▸).

A strategy to avoid the requirement for protein purification, and the challenges and optimization associated with it, is to use fractionated or crude cell lysates. This approach has been used to identify cytosol composition and cytosolic complexes also utilizing visual proteomics approaches (Ho *et al.*, 2020 ▸; Tüting *et al.*, 2021 ▸; Skalidis *et al.*, 2022 ▸; Cooney *et al.*, 2023 ▸). Yet, with such approaches information about the native environment is largely lost due to lysis. This underscores the need for tailored preparation protocols to allow SPA data collection while preserving the native environment as much as possible.

An elegant way of circumventing the need for target protein isolation or *in vitro* reconstitution is to image the target in its native environment inside the cell. Cryo-electron tomography (cryo-ET), in which a tilt series of images is acquired and then reconstructed into a three-dimensional volume, is used more and more frequently to solve structures of proteins *in situ* (Young & Villa, 2023 ▸). Often this requires sample thinning by cryo-focused ion beam (cryo-FIB) milling, a technology that enables the generation of typically 80–300 nm thin slices (‘lamellae’) of vitrified cells or tissue, to reach a sample thickness compatible with analysis by transmission electron microscopy (for comprehensive reviews on different strategies of *in situ* cryo-ET, see Young & Villa, 2023 ▸; Berger, Premaraj *et al.*, 2023 ▸). The true value of cryo-FIB milling and cryo-ET, combined with other integrative structural biology strategies, is clearly the ability to provide structural information in a cellular context, thereby ‘bridging structural and cell biology’ (McCafferty *et al.*, 2024 ▸; Nogales & Mahamid, 2024 ▸; Kyrilis *et al.*, 2024 ▸). More recent developments established cryo-FIB milling and cryo-ET from tissue or even whole organisms, opening up a whole new world for structural biologists (Tamborrini *et al.*, 2023 ▸; Schiøtz *et al.*, 2024 ▸; Matsui *et al.*, 2024 ▸; Creekmore *et al.*, 2024 ▸). Applying cryo-FIB milling and cryo-ET to cellular samples or tissue, however, holds a few challenges of its own. For instance, sample throughput and data collection often remain a limiting factor, although new hardware developments for cryo-FIB lamellae preparation and recent advances in automation of tilt-series acquisition have enabled the collection of larger data sets in significantly less time (Eisenstein *et al.*, 2023 ▸, 2024 ▸). Limitations in sample throughput may lead to the requirement for the particles of interest to be naturally abundant and distinguishable in the cell to allow high-resolution subtomogram averaging (STA). This is of particular importance considering that only a fraction of a cell is contained in a single lamella (Berger, Premaraj *et al.*, 2023 ▸). Thanks to major advances in software development, STA is becoming more powerful and streamlined. However, several challenges such as the comparably low signal-to-noise ratio inherent to tilt series, missing wedge information and accurate tilt-series alignment still impact the achievable resolution of this approach (Pyle & Zanetti, 2021 ▸; Zivanov *et al.*, 2022 ▸; Burt *et al.*, 2024 ▸; Förster & Briegel, 2024 ▸). Although achieving subnanometre resolution by *in situ* STA may become achievable for molecules in the 100 kDa range, it is currently still mostly limited to large molecular assemblies, including ribosomes, viral capsids, carboxysomes and RuBisCO (Sutton *et al.*, 2020 ▸; Ni, Frosio *et al.*, 2022 ▸; Russo *et al.*, 2022 ▸; Berger, Dumoux *et al.*, 2023 ▸; Evans *et al.*, 2023 ▸; Ni, Sun *et al.*, 2022 ▸; Sun *et al.*, 2024 ▸).

To overcome the aforementioned limitations, hybrid strategies have been experimented. These includes combining the collection of a high-dose image and a low-dose tilt series to achieve subnanometre resolution *in situ* (Song *et al.*, 2020 ▸; Sanchez *et al.*, 2020 ▸). Other studies establish SPA pipelines to be directly applied to tilt series or combine SPA data collection of crowded samples with innovative particle-localization strategies to allow *in situ* SPA (Cheng *et al.*, 2021 ▸; Lucas *et al.*, 2022 ▸, 2023 ▸; Liu *et al.*, 2023 ▸). Examples employing such strategies have demonstrated their feasibility; however, they often still require combination with cryo-ET to obtain suitable templates for particle picking (You *et al.*, 2023 ▸; Zhang *et al.*, 2024 ▸). Moreover, all of the above-described strategies require the samples to be naturally thin or to be prepared by cryo-FIB milling to enable SPA or hybrid data collection (Guaita *et al.*, 2022 ▸).

**Figure 1:**
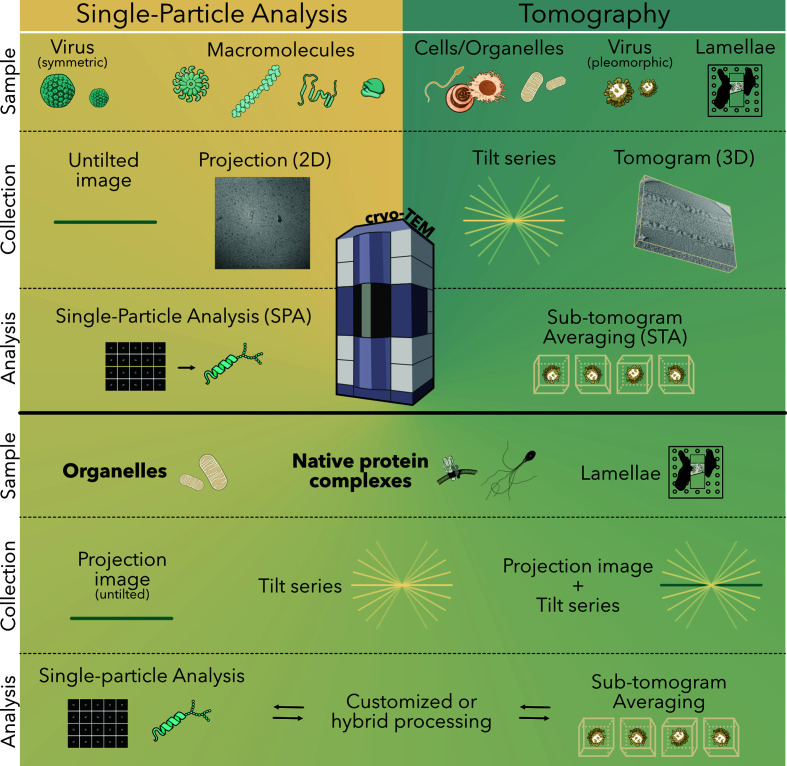
Cryo-EM single-particle analysis (SPA) and cryo-ET subtomogram averaging (STA) classically have defined sample preparation, data collection and processing (upper panel). Recent studies demonstrate that the boundaries between the techniques are blurring (lower panel). (Multiple illustrations from NIAID NIH BIOART Source (https://bioart.niaid.nih.gov).

Recent studies demonstrate that the boundaries between cryo-EM/SPA and cryo-ET/STA are starting to blur (Fig. 1 ▸) as the sample types and pipelines used in both techniques increasingly overlap. The studies highlighted in this review impressively demonstrate that it is possible to take advantage of SPA to achieve high resolution while maintaining near-native conditions. To achieve this, tailored strategies are used to minimally isolate the biological structure of interest directly from cells to allow SPA data collection. Of note, the target structures of the examples below are naturally abundant and the constrained spatial organization of the target within the cell was instrumental in developing a tailored isolation strategy. The selected studies nicely exemplify how such tailored sample-preparation strategies can enable direct data collection for SPA to gain access to high-resolution structural information of molecular assemblies that are extremely difficult to reconstitute. At the same time, they demonstrate how the range of samples suitable for SPA is expanding towards thicker and more complex samples, increasing the extent to which the target environment can be maintained.

## 2. SPA for *de novo* identification of proteins in complex molecular assemblies

**Figure 2:**
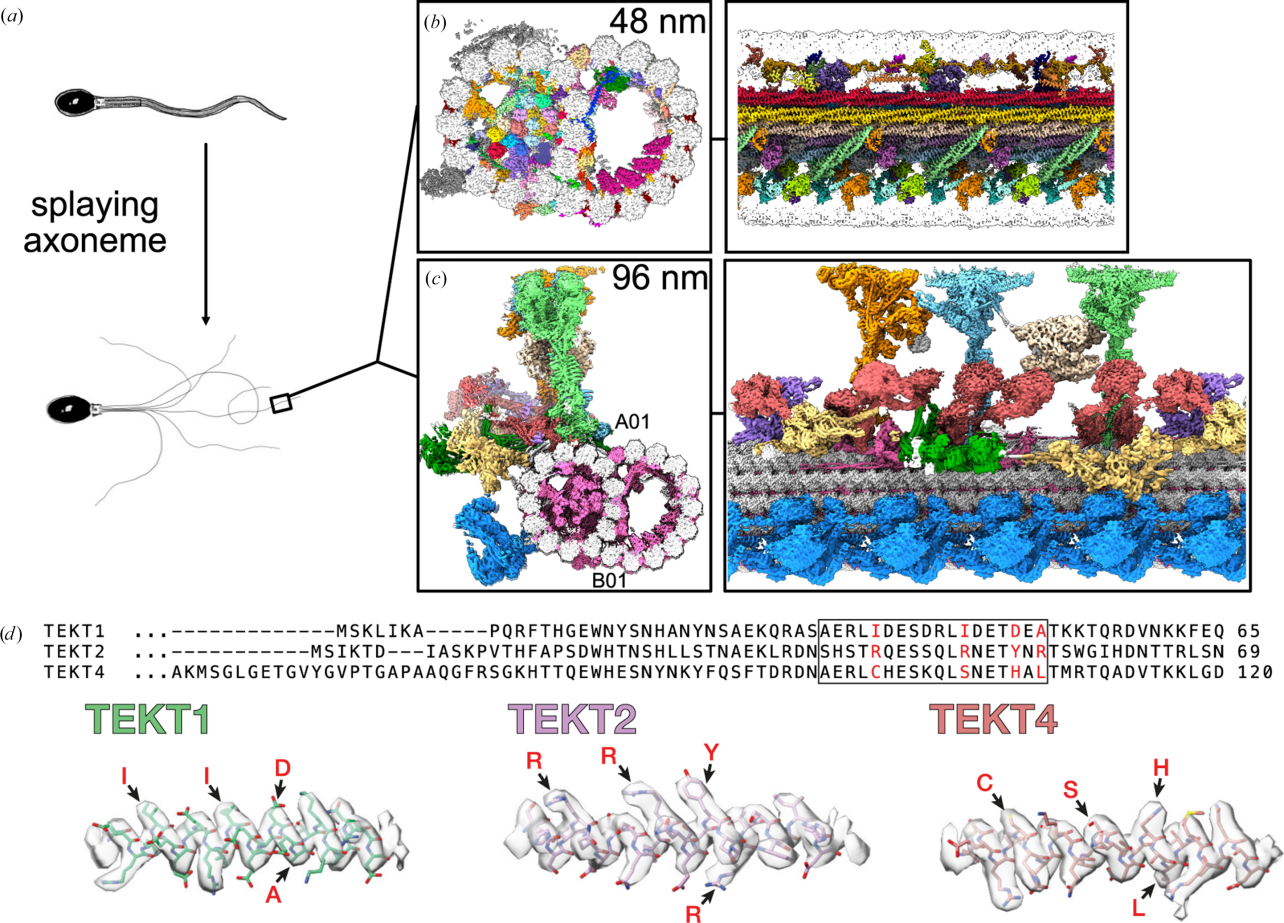
*De novo* identification of proteins with SPA. (*a*) Brief summary of the sample-preparation strategy applied to prepare bovine sperm doublet microtubules for SPA data collection. (*b*) Two orthogonal views of the 48 nm repeat of axonemal doublet microtubules from bovine sperm, highlighting all the microtubule inner proteins identified (PDB entry 8otz). (*c*) Two orthogonal views of the 96 nm repeat of axonemal doublet microtubules showing all the external complexes (PDB entry 9fqr). (*d*) Representative densities of three different microtubule inner proteins (Tektin-1, Tektin-2 and Tektin-4) and their corresponding sequences, illustrating how the protein sequence can be ‘read’ from the density.

When atomic or near-atomic (<3.5 Å) resolution is achieved by SPA, no prior knowledge is needed to identify the protein of interest. The map contains all of the information needed to ‘read’ the protein sequence (Chojnowski *et al.*, 2022 ▸; Jamali *et al.*, 2024 ▸). In several recent studies, SPA has been used as a discovery tool to identify many of the components of the axoneme and its intricate assembly (Fig. 2 ▸; Gui *et al.*, 2021 ▸; Gui, Wang *et al.*, 2022 ▸; Leung *et al.*, 2023 ▸, 2025 ▸; Zhou *et al.*, 2023 ▸). Of note, earlier studies applied extensive purification protocols involving deflagellation and multiple centrifugation steps (Gui *et al.*, 2021 ▸; Gui, Wang *et al.*, 2022 ▸), whereas tailored sample preparation was key in more recent studies (Leung *et al.*, 2023 ▸, 2025 ▸; Zhou *et al.*, 2023 ▸). Sperm cells were simply demembranated and the mitochondrial sheath removed by DTT. Addition of ATP induced microtubule sliding, as shown in earlier studies (Lindemann *et al.*, 1980 ▸). Since the membrane and mitochondrial sheath have been removed and thus no longer restrain the axoneme in a confined space, sliding results in splaying of the axonemal doublet microtubules (DMTs; Fig. 2 ▸*a*). The splayed axonemes, still attached to the sperm head, were then directly applied onto an EM grid and plunge-frozen for SPA data collection. The elegance of this protocol lies in its simplicity, involving only two steps of incubation in straightforward buffer compositions, while maintaining the integrity not only of the DMT scaffold but also most associated structures.

The axoneme is a huge molecular assembly comprised of nine DMTs surrounding a central pair of singlet microtubules. It constitutes the central element of cellular flagella and has been the subject of many structural studies (Satir & Christensen, 2007 ▸; Ishikawa, 2017 ▸; Ma *et al.*, 2020 ▸; Walton *et al.*, 2021 ▸, 2023 ▸). In many of these studies, microtubule inner proteins (MIPs) and microtubule-associated proteins (MAPs) were shown to fill the lumen of the microtubules and decorate their lattice (Owa *et al.*, 2019 ▸; Ma *et al.*, 2019 ▸; Gui *et al.*, 2021 ▸; Leung *et al.*, 2021 ▸; Gui, Croft *et al.*, 2022 ▸; Kubo *et al.*, 2023 ▸). The MIP and MAP repertoires were shown to be cell type-specific or even to change per subcellular location (Leung *et al.*, 2021 ▸). The extent to which axonemal microtubules are decorated and the identity of the decorating proteins have largely remained elusive. Due to this gap in knowledge, overexpression and purification or computational modelling is practically impossible. Circumventing these knowledge-based methods requires structural analysis either by *in situ* analysis (*i.e.* cryo-FIB and cryo-ET) or in a setting that preserves the native molecular decoration of the DMT and enables SPA. The latter was achieved by application of the tailored protocol described above, resulting in high-resolution structures of sperm axonemal DMTs from different species, and the identification of a multitude of associated proteins (Leung *et al.*, 2023 ▸, 2025 ▸; Zhou *et al.*, 2023 ▸).

Data collection of such samples differs from standard SPA data collection. The structure of interest is distributed less regularly across the grid, complicating automated data collection. The positions for image acquisition are defined by tracing the shape of the splayed DMTs across the grid. This was carried out semi-automatically in *SerialEM* (Mastronarde, 2005 ▸). Due to the complexity of this near-native sample and the sheer number of proteins bound to the DMT, the processing had to be carried out in multiple sequential steps (for details, see the original publications: Leung *et al.*, 2023 ▸, 2025 ▸). The quality of the resulting maps was sufficiently high to unambiguously identify most of the proteins by ‘reading’ the sequence from the density (Fig. 2 ▸*d*).

These studies revealed that sperm axonemal DMTs are the most specialized, with epithelial cilia having only minor differences across tissues. For example, sperm DMT anchors a T-complex protein ring complex (TRiC) chaperone every 96 nm that may contribute to construction or maintenance of the long flagella of mammalian sperm. Resolving the structure of radial spoke 3 revealed the binding sites of kinases associated with regeneration of ATP and regulation of ciliary motility. The large data set enabled axonemal dyneins to be captured in their prestroke states, illuminating conformational changes that occur during ciliary movement. Taken together, these studies revealed how elements of chemical and mechanical regulation are embedded within the axoneme and provide valuable resources for understanding the aetiology of ciliopathy and infertility.

Apart from significantly advancing the mechanistic understanding of axonemal DMT structures, these studies are a major achievement in structural biology. The 96 nm repeat is composed of >180 proteins with >0.5 million residues and >4 million atoms. Reconstitution of such a complex structure seems almost impossible. The sheer number of proteins identified directly from the electron-density maps highlights the power of SPA, on near-native samples, as a discovery tool. Although the structure was obtained from a splayed axoneme, all MIPs and MAPs identified remained in the preparation, as demonstrated in the original publication by comparing with a STA structure. The strategy of splaying the axoneme to obtain high-resolution structures of DMTs by SPA could further be transferred to axonemes of motile or primary cilia from different cell types and organisms.

## 3. SPA of membrane proteins in native vesicles

Structure determination of membrane proteins has highly benefited from SPA (Nygaard *et al.*, 2020 ▸; Piper *et al.*, 2022 ▸). Many of these structures are of membrane proteins solubilized in detergents (Sgro & Costa, 2018 ▸; Piper *et al.*, 2022 ▸; Vénien-Bryan & Fernandes, 2023 ▸). The desire to resolve the structures in the lipidic environment led to the application of nanodisc technologies. Nanodiscs can either be protein or amphipathic polymer ‘belts’ that hold together discoidal lipid bilayers into which detergent-solubilized membrane proteins can be reconstituted (Sgro & Costa, 2018 ▸; Piper *et al.*, 2022 ▸; Vénien-Bryan & Fernandes, 2023 ▸). The size of these nanodiscs (8–16 nm) makes them ideal for SPA; however, the membrane proteins still need to be isolated from their native membranes. Structural characterization of membrane proteins on native vesicles or microsomes has been widely successful with cryo-ET (Zeev-Ben-Mordehai *et al.*, 2014 ▸; Vollmer *et al.*, 2020 ▸; Gemmer *et al.*, 2023 ▸; Tringides *et al.*, 2023 ▸; Pyle *et al.*, 2025 ▸), but has been deemed inappropriate for SPA, due to the size of the vesicles leading to overlapping information in the projection images.

Two recent studies proved that the structure of V-ATPase can be resolved by SPA directly on synaptic vesicles (Wang *et al.*, 2024 ▸; Coupland *et al.*, 2024 ▸). Mammalian vacuolar- or vesicular-ATPases (V-ATPases) are membrane-embedded assemblies of 16 proteins which use ATP hydrolysis to facilitate pH control in different compartments via proton translocation (Forgac, 2007 ▸). Synaptic vesicles (SV), organelles dedicated to the controlled release of neurotransmitters into the synapse between an axon terminal and a dendrite, utilize the proton gradient produced by V-ATPase for the directional uptake of these neurotransmitters (Volknandt, 1995 ▸; Südhof, 2004 ▸). Loaded vesicles can release neurotransmitters into the synapse by membrane fusion and can afterwards be recycled for reloading (Volknandt, 1995 ▸). Many structures of individual or multiple building blocks of V-ATPase, or of the entire assembly, have been determined by SPA and crystallography (Vasanthakumar & Rubinstein, 2020 ▸). Thus far, all structures were obtained from reconstituted or solubilized and purified V-ATPase. As for the example of axonemal DMTs, reconstitution is limited to components that have already been identified in the target structure. In solubilizing V-ATPase complexes key contextual information is lost, such as the native membrane environment and putatively bound inter­action partners.

**Figure 3:**
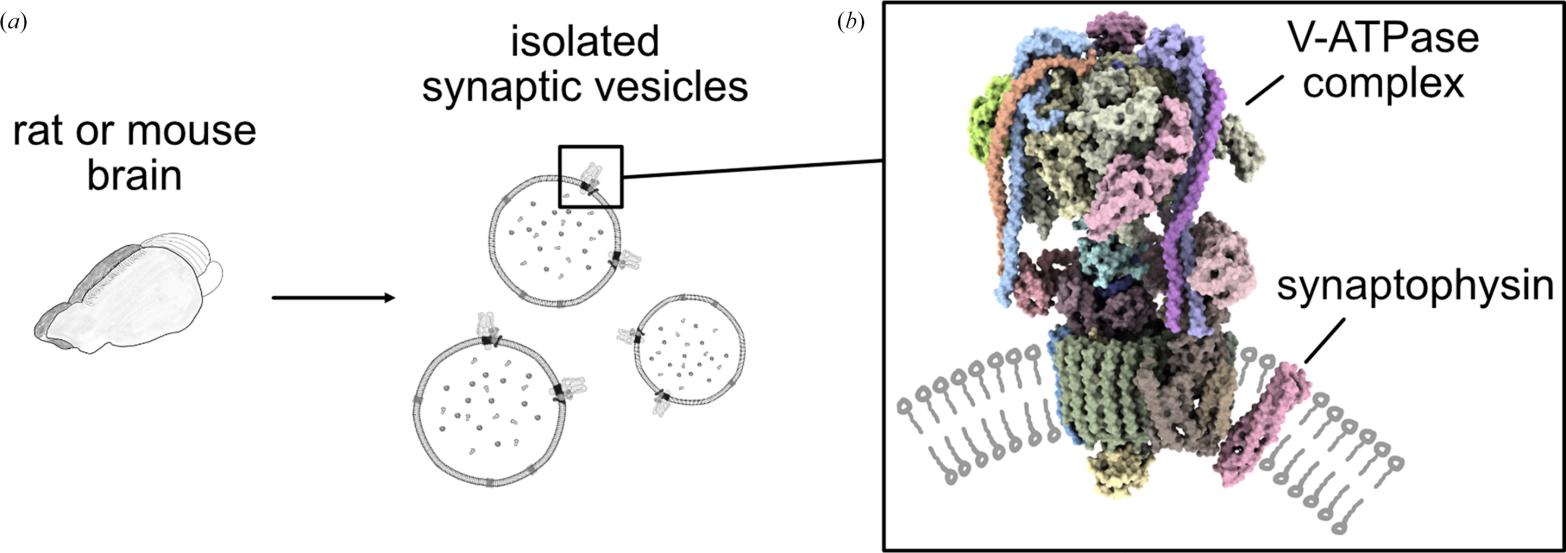
High-resolution structure of V-ATPase in native synaptic vesicles. (*a*) Sample preparation (Coupland *et al.*, 2024 ▸; Wang *et al.*, 2024 ▸). (*b*) Structure of the V-ATPase complex bound to the newly described interaction partner synaptophysin (PDB entry 9brb). Schematic lipids (grey) were added manually.

In the two recent studies, SVs were isolated and enriched, and the V-ATPase structures were determined with SPA without solubilizing the complexes (Wang *et al.*, 2024 ▸; Coupland *et al.*, 2024 ▸). The protocols used to isolate and enrich SVs were based on affinity purification (Fig. 3 ▸*a*; Chantranupong *et al.*, 2020 ▸; Abbas *et al.*, 2020 ▸; Leitz *et al.*, 2024 ▸). Mass spectrometry confirmed the isolated vesicles to be SVs and the functionality of V-ATPase complexes were assayed by testing intravesicular acidification (Coupland *et al.*, 2024 ▸) or by Ca^2+^-triggered fusion assays (Wang *et al.*, 2024 ▸). Data collection and processing on the SVs followed common SPA protocols. Using heterogeneous refinement, three rotational states of V-ATPase were resolved, which were further improved upon with non-uniform refinement (Punjani *et al.*, 2020 ▸). This resulted in maps at resolutions of 3.6, 3.9 and 3.5 Å of rotational states 1, 2 and 3, respectively (Coupland *et al.*, 2024 ▸), or 4.3 Å for all three rotational states (Wang *et al.*, 2024 ▸).

The high resolution of these maps enabled analysis of the native membrane environment of the target structure and the identification of an endogenous interaction partner, synaptophysin, which was not observed in previous structures from reconstituted or solubilized V-ATPase complexes (Fig. 3 ▸*b*). *AlphaFold* models of proteins identified in mass-spectrometry data of SVs were fitted into the additional density that could not be explained by any of the V-ATPase components. Out of these models, only synaptophysin and synaptoporin fit into the associated density with high confidence. At the resolution reached, it was not possible to fully resolve the interacting protein. Yet, high-resolution density at the interface to V-ATPase strongly suggested the binding partner to be synaptophysin (Fig. 3 ▸*b*; Coupland *et al.*, 2024 ▸). Structural analysis of synaptophysin knockout-derived vesicles further supported specific binding of synaptophysin and not synaptoporin to V-ATPase (Wang *et al.*, 2024 ▸). Of note, a third study applied cryo-ET to similarly isolated vesicles, resolving V-ATPase at a resolution of 16.7 Å and, utilizing integrative structural biology tools, also identified synaptophysin as an interaction partner (Kravčenko *et al.*, 2024 ▸). Importantly, the identification of synaptophysin was only possible due to structure determination of V-ATPase in native synaptic vesicles, as earlier studies using detergent-solubilized V-ATPase did not indicate this interaction (Wang *et al.*, 2020 ▸; Abbas *et al.*, 2020 ▸). Synaptophysin has been associated with a subtle phenotype of intellectual disability in humans and learning deficits in mice (Tarpey *et al.*, 2009 ▸; Schmitt *et al.*, 2009 ▸). Additionally, knockout mice showed significantly increased susceptibility to seizures induced by kainic acid stress (Wang *et al.*, 2024 ▸). In using a more native state, the studies were able to capture this interaction, which may shed light on specific disease pathways that otherwise would have remained unknown.

Apart from the additional synaptophysin density, the structures showed cholesterol to assemble in a regularly spaced manner around the proton-conducting c-ring of V-ATPase and evidence of post-translational modification (Coupland *et al.*, 2024 ▸). SV membranes are naturally rich in cholesterol, and V-ATPase function requires the presence of cholesterol (Zhang *et al.*, 2010 ▸). Although previous work identified similar densities around the c-ring, the identities of these were unclear due to the purification protocol making use of a sterol-derived detergent, glycol-diosgenin, which at this resolution is indistinguishable from cholesterol (Vasanthakumar *et al.*, 2019 ▸). Finally, additional density was found on certain residues previously shown to be glycosylated (Wang *et al.*, 2020 ▸). Consistent with the previous study, only the first two polysaccharides were found to be ordered (Coupland *et al.*, 2024 ▸). An additional putative glycosylation site was identified at Asn339 (Wang *et al.*, 2024 ▸). Identification of these previously undiscovered structural details again highlights the impact of maintaining the target protein in a near-native context, the vesicle membrane, for protein structure determination.

Overall, preserving the native membrane environment of the V-ATPase complexes provided novel insights into binding partners that would remain unidentified when using solubilization-based approaches. The SV isolation strategies applied allowed the data to be collected and processed following largely standard SPA protocols with optimized data-processing strategies. Importantly, the resulting maps were sufficient to build atomic models, highlighting that data collected on vesicles containing a multitude of nontarget proteins can be used to obtain high-resolution structures of proteins while embedded in their native membrane. In the future, similar strategies could be applied to proteins integral to vesicle membranes or contained within vesicles.

## 4. SPA of super-complexes in intact organelle membranes

Cellular organelles are studded with membrane proteins and, as discussed in the previous section, maintaining their native environment is instrumental to identifying the native structural conformation, native interaction partners and the membrane composition around these proteins. A recent study resolved the respiratory-chain complexes in intact mitochondria isolated from pig heart, demonstrating that SPA can be applied to these organelles directly (Zheng *et al.*, 2024 ▸). Preserving the membrane environment and obtaining structural insights without solubilization of the target complex enabled visualization of the stoichiometry of the electron-transport chain (ETC) complexes. More impressively, ETC complexes were resolved at resolutions better than 3 Å within intact mitochondria, densely packed with other proteins, membranes and metabolites.

**Figure 4:**
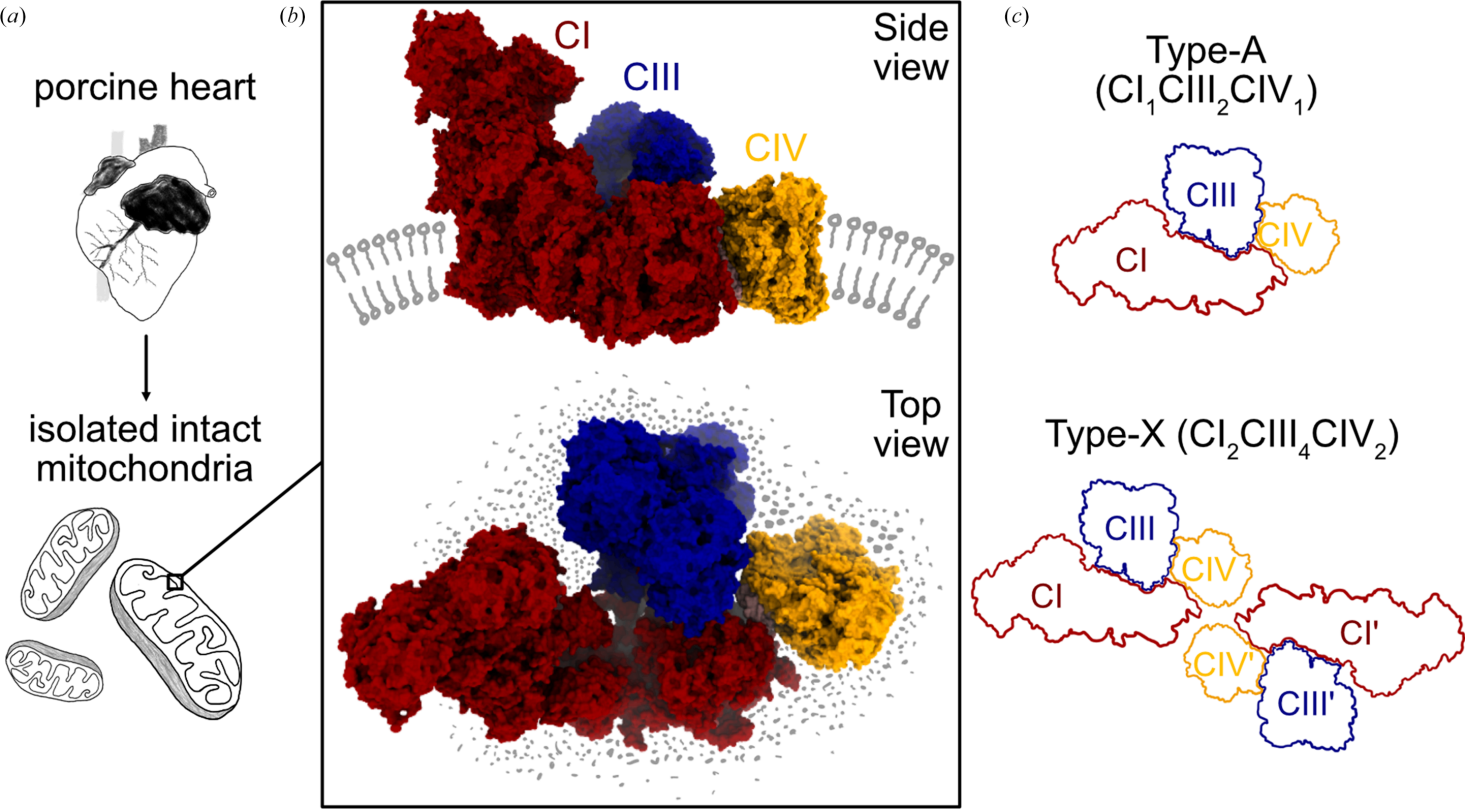
Structure of respiratory supercomplexes from intact porcine mitochondria. (*a*) Sample preparation. (*b*) Structure of type-A (CI_1_CIII_2_CIV_1_) SCs from intact mitochondria (PDB entry 8ugi). Schematic lipids (grey) indicating the native membrane environment were added manually. (*c*) Schematic comparison of the stoichiometry of the common type-A and previously undescribed type-X supercomplexes.

Mitochondria are central for the generation of ATP in most eukaryotic cells. The machinery behind this biochemical process is comprised of multiple proteins embedded in the inner mitochondrial membrane, which have been demonstrated to assemble into supercomplexes (SCs; Schägger & Pfeiffer, 2000 ▸). The three main building blocks of these SCs, complex I (CI, NADH:ubiquitine oxidoreductase), complex III (CIII, ubiquinol-cytochrome *c* oxidoreductase) and complex IV (CIV, cytochrome *c* oxidase), facilitate proton translocation into the intermembrane space, maintaining a proton gradient across the inner mitochondrial membrane that drives ATP synthesis through F_1_F_0_-ATPase (Milenkovic *et al.*, 2017 ▸). CI, CIII and CIV form a so-called respirasome with different stoichiometries. The most extensively studied stoichiometry is CI/CIII_2_/CIV (type-A). Ubiquinone/ubiquinol (coenzyme Q, Q/QH_2_) and cytochrome *c* (cyt *c*) are important cofactors for respirasomes, shuttling electrons between the different parts of the complexes (Milenkovic *et al.*, 2017 ▸). Multiple SPA structures have provided detailed structural and mechanistic insight into different complexes and SC assembly (Zhu *et al.*, 2016 ▸; Letts *et al.*, 2016 ▸, 2019 ▸; Wu *et al.*, 2016 ▸; Guo *et al.*, 2017 ▸; Blaza *et al.*, 2018 ▸; Kampjut & Sazanov, 2020 ▸; Spikes *et al.*, 2021 ▸; Agip *et al.*, 2023 ▸; Bridges *et al.*, 2023 ▸; Lai *et al.*, 2023 ▸; Du *et al.*, 2023 ▸; Mühleip *et al.*, 2023 ▸). The studies above purified the respiratory complexes or SCs, often utilizing solubilization approaches. In the study highlighted here, mitochondria were isolated from porcine hearts and directly plunge-frozen without further treatment or preparation (Fig. 4 ▸*a*).

Data for reconstruction of SCs in intact mitochondria was collected according to common SPA protocols, but data processing was customized substantially. Three major challenges hampered high-resolution reconstruction. Firstly, since whole mitochondria were imaged, the samples were thick, leading to low signal-to-noise ratio and high defocus variations. Secondly, the particles were situated in a densely crowded environment, complicating particle identification and alignment. Finally, due to the SCs being embedded in the membrane, initial alignments were dominated by membrane signal and protein densities were not well defined. Therefore, a customized processing approach was developed to achieve high-resolution averages. For example, as SC stoichiometry in native settings was previously not well defined, three strategies for initial reference generation were employed to ensure that unbiased maps could be obtained. These strategies included, firstly, the use of subvolume averages from cryo-ET data collected on the same sample and, secondly, *ab initio* reconstruction from 2D classes containing clear CIII_2_ membrane features. In a third strategy an *ab initio* reference was reconstructed from particles after weakening the membrane signal, since the strong signal from surrounding membranes can significantly interfere with *ab initio* reconstruction, 2D and 3D classification as well as refinement (Zheng *et al.*, 2024 ▸). Applying these strategies for 3D classification, particles with clear features of type-A SCs (CI/CIII_2_/CIV) were identified. Further classification and refinement steps revealed additional densities around the type-A SC, indicating the presence of other SC stoichiometries. Following multiple rounds of cross-classification and local refinement, multiple SC types were identified, of which four were included in further refinement: CI/CIII_2_/CIV (type-A), CI/CIII_2_/CIV_2_ (type-B), CI_2_/CIII_2_/CIV_2_ (type-O) and CI_2_/CIII_4_/CIV_2_ (type-X) (Figs. 4 ▸*b* and 4 ▸*c*). Multilevel local refinement and masking of subareas of the complexes resulted in an overall resolution of ∼2.5 Å and local resolutions of up to 1.8 Å for type-A SCs.

The high resolution of the maps of different SC subtypes allowed a detailed analysis of interaction interfaces. While clear interfaces between CI and CIII_2_, as well as between CI and CIV, could be identified, no direct protein interactions were found between CIII_2_ and CIV. Lipids populating the space between the different components further seem to mediate interaction and thus contribute to SC formation. Since the native state of the mitochondrial membrane architecture was largely preserved and the density maps of the membrane regions were extremely clear, modelling membranes and subsequent analysis of the membrane geometry was also possible. These observations again highlight the advantage of imaging target structures in a native setting, as otherwise effects on membrane curvature would likely remain undiscovered. The maps further enabled a clear analysis of the designated coenzyme-Q-binding site in CI and identification of five distinct coenzyme-Q-binding states. Analysis of native SCs after different ischemia-mimicking treatments led to the identification of potential transition states of CI that had not previously been described.

Although the physiological relevance of SC assemblies is still a matter of debate, the newly identified stoichiometries and interaction interfaces further advance our understanding of potential electron shuttling through SCs, and importantly provides the first structural evidence of SC conformations in native membranes (Blaza *et al.*, 2018 ▸; Chung *et al.*, 2022 ▸; Milenkovic *et al.*, 2023 ▸). Of note, a complementary recent study revealed an *in situ* structure of a respiratory SC from mitochondria in intact *C. reinhardtii* cells. Here, the *in situ* data were obtained by a combined cryo-FIB milling and cryo-ET approach, resolving the respiratory SC at an impressive overall resolution (∼5.4 Å) and resolving yet a different stoichiometry compared with those described above (Waltz *et al.*, 2025 ▸). Additionally, supercomplexes were purified from isolated mitochondria to allow SPA data collection and analysis resulting in a 2.8 Å resolution structure. This further exemplifies the current advantage of SPA over STA in terms of the achievable resolution. At the same time, STA in combination with cryo-FIB milling can reveal true *in situ* structures. Finally, the work highlighted in this section is an excellent example of maintaining the structure of interest in its near-native state (*i.e.* the mitochondrial membrane within intact mitochondria) while achieving high resolution by directly applying SPA (Zheng *et al.*, 2024 ▸). This study further highlights the potential of SPA to obtain high-resolution structures of organelle-embedded membrane-proteins.

## 5. The expanding range of increasingly native samples suitable for SPA

Each study summarized above highlights different advantages of applying SPA to increasingly native samples and constitutes a breakthrough for SPA. The case of sperm axonemal DMTs exemplifies cases where the complexity of the target macromolecular assembly would be nearly impossible to reconstitute (Leung *et al.*, 2023 ▸, 2025 ▸). The V-ATPase on synaptic vesicles demonstrates that keeping the native environment can be crucial to capture interacting partners (Coupland *et al.*, 2024 ▸; Wang *et al.*, 2024 ▸). Resolving the respirasome in intact mitochondria proved that SPA could achieve high-resolution structures from intact organelles, despite their thickness, thus substantially expanding the range of samples that can be used for SPA.

Common to these three examples is that reconstitution and purification was avoided and instead the structure of interest was isolated directly from cells, while maintaining their native state and, to a certain degree, their native environment. This allowed the discovery of previously undetected interaction partners or complex stoichiometries. Destabilization or loss of associated components are known artefacts in isolating large macromolecular assemblies from their native environment. This is especially important to consider in cases where the composition of the target complex is not entirely clear. Loss of an associated protein may remain completely undetected if the presence of that protein is not known or expected in the first place. The studies summarized above are examples in which tailored sample-preparation strategies manage to reduce the biological system of interest to allow SPA data collection, while the structural integrity of the target and interaction partners is maintained.

As the range of samples amenable to SPA is expanding, sample-preparation requirements are changing as well. This will open new possibilities to achieve high-resolution structures of targets within their native environment that were previously unattainable through SPA.

## 6. Concluding remarks

With both hardware and software continuing to improve, we predict that SPA on more native samples will gain importance as an approach to resolve large macromolecular assemblies at high resolution. In addition to allowing determination of the exact composition of these assemblies in a native or near-native setting, the high resolution reached by SPA may enable *de novo* protein identification. Being able to apply SPA to thicker and more complex native samples holds great promise to fill current gaps in structural information, especially for large protein complexes that are difficult to reconstitute. As the range of samples is now expanding to samples as complex as organelles, SPA further closes the gap to *in situ* information, typically requiring cryo-FIB milling and cryo-ET.

As the boundaries between cryo-EM/SPA and cryo-ET/STA are blurring (Fig. 1 ▸), we would like to suggest viewing cryo-EM as a single technology with a spectrum of potential approaches. The term spectrum seems fitting, as we believe it captures the range of different sample-preparation, data collection and processing strategies that have already been applied and that are still being developed. Currently, a common theme is that cryo-EM/SPA and cryo-ET/STA are used in a complementary manner. Higher resolution is still more commonly reached by SPA, increasing the level of confidence when identifying proteins directly from density maps. Yet, cryo-ET/STA can provide true *in situ* insights and can indicate whether components of the target structure were lost during sample preparation for SPA. Thus, many studies, including those beyond the examples highlighted here, combine both approaches to complement each other. Thereby, they obtain high-resolution, *in situ* contextual information on a variety of macromolecules, including viral particles, filamentous structures, respiratory supercomplexes, carboxysomes and RuBisCO (Ni, Sun *et al.*, 2022 ▸; Kravčenko *et al.*, 2024 ▸; Coupland *et al.*, 2024 ▸; Wang *et al.*, 2024 ▸; Zheng *et al.*, 2024 ▸; Sun *et al.*, 2024 ▸; Leung *et al.*, 2025 ▸). As another alternative, cryo-FIB milling and SPA on lamellae have also achieved high-resolution structures (Lucas *et al.*, 2022 ▸, 2023 ▸; You *et al.*, 2023 ▸).

Ultimately, the biological system and the research question will need to be considered when deciding on the strategy required to obtain structural information. If high-resolution structural information is the goal, SPA on native samples may be a valuable alternative to obtain such structural information and, to a certain extent, contextual information on the native protein environment. The extent to which these samples can be both complex and native will continue to be redefined as the capabilities of SPA evolve and improve.
